# The European reference network for metabolic diseases (MetabERN) clinical pathway recommendations for Pompe disease (acid maltase deficiency, glycogen storage disease type II)

**DOI:** 10.1186/s13023-024-03373-w

**Published:** 2024-11-01

**Authors:** Giancarlo Parenti, Simona Fecarotta, Marianna Alagia, Federica Attaianese, Alessandra Verde, Antonietta Tarallo, Vincenza Gragnaniello, Athanasia Ziagaki, Maria Jose’ Guimaraes, Patricio Aguiar, Andreas Hahn, Olga Azevedo, Maria Alice Donati, Beata Kiec-Wilk, Maurizio Scarpa, Nadine A. M. E. van der Beek, Mireja Del Toro Riera, Dominique P. Germain, Hidde Huidekoper, Johanna M. P. van den Hout, Ans T. van der Ploeg, and the MetabERN Subnetwork for Lysosomal Disorders, Ivo Baric, Spyros Batzios, Nadia Belmatoug, Andrea Bordugo, Annet M. Bosch, Anais Brassier, Alberto Burlina, David Cassiman, Brigitte Chabrol, Efstathia Chronopoulou, Maria Luz Couce-Pico, Niklas Darin, Anibh M. Das, Francois G. Debray, Patrick Deegan, Luisa M. de Abreu Freire Diogo Matos, Javier De Las Heras Montero, Maja Di Rocco, Dries Dobbelaere, Francois Eyskens, Ana Ferreira, Ana M. Gaspar, Serena Gasperini, Antonio González-Meneses López, Salvatore Grosso, Nathalie Guffon-Fouilhoux, Julia Hennermann, Tarekegn G. Hiwot, Simon Jones, Sandra Kingma, Veroniki Komninaka, Elena Martín-Hernández, Esmeralda Martins, Diana Miclea, György Pfliegler, Esmeralda Rodrigues, Dariusz Rokicki, Dominique Roland, Frank Rutsch, Alessandro Salviati, Ivailo Tournev, Kurt Ullrich, Peter M. van Hasselt, Suresh Vijay, Natalie Weinhold, Peter Witters, Jiri Zeman

**Affiliations:** 1 MetabERN Subnetwork for Lysosomal Disorders, Rotterdam, The Netherlands; 2 Telethon Institute of Genetics and Medicine, Via Campi Flegrei 34, Pozzuoli, Naples, Italy; 3 Department of Translational Medical Sciences, University of Naples Federico II, Via S. Pansini 5, Naples, Italy; 4 Azienda Ospedaliera Universitaria Federico II, Naples, Italy; 5 Department of Endocrinology and Metabolism, Center of Excellence for Rare Metabolic Diseases in Adults, Charite-Universitätsmedizin Berlin, Berlin, Germany; 6 Pneumology Department, Reference Center on Lysosomal Storage Disorders, Hospital Senhora da Oliveira, Guimarães, Portugal; 7 Clinica Universitaria de Medicina I, Universidade de Lisboa, Lisbon, Portugal; 8 Department of Child Neurology, Justus-Liebig University, Giessen, Germany; 9 Cardiology Department, Reference Center on Lysosomal Storage Disorders, Hospital Senhora da Oliveira, Guimarães, Portugal; 10 Life and Health Sciences Research Institute (ICVS), School of Medicine, University of Minho, Braga, Portugal; 11 ICVS/3Bs PT Government Associate Laboratory, Braga/Guimarães, Portugal; 12 Metabolic and Neuromuscular Unit, Meyer Children Hospital-University of Florence, Florence, Italy; 13 Unit of Rare Metabolic Diseases, Jagiellonian University Medical College, Kraków, Poland; 14 The John Paul II Specjalist Hospital in Kraków, Kraków, Poland; 15 Centro Coordinamento Regionale Malattie Rare, Azienda Sanitaria Universitaria del Friuli Centrale, Udine, Italy; 16 Center for Lysosomal and Metabolic Diseases, Erasmus MC, Erasmus University Medical Center, Rotterdam, Netherlands; 17 Metabolic Unit, Department of Pediatric Neurology, Hospital Universitario Vall d’Hebron Barcelona, Barcelona, Spain; 18 Division of Medical Genetics, University of Versailles, Montigny, France; 19 Department of Pediatrics, Center for Lysosomal and Metabolic Diseases, Erasmus MC University Medical Center, Rotterdam, The Netherlands

**Keywords:** Pompe disease, Glycogen storage disease (GSD) type II, Acid alpha-glucosidase deficiency, Acid maltase deficiency, Lysosomal storage disease

## Abstract

Clinical pathway recommendations (CPR) are based on existing guidelines and deliver a short overview on how to deal with a specific diagnosis, resulting therapy and follow-up. In this paper we propose a methodology for developing CPRs for Pompe disease, a metabolic myopathy caused by deficiency of lysosomal acid alpha-glucosidase. The CPR document was developed within the activities of the MetabERN, a non-profit European Reference Network for Metabolic Diseases established by the European Union. A working group was selected among members of the MetabERN lysosomal storage disease subnetwork, with specific expertise in the care of Pompe disease, and patient support group representatives. The working strategy was based on a systematic literature search to develop a database, followed by quality assessment of the studies selected from the literature, and by the development of the CPR document according to a matrix provided by MetabERN. Quality assessment of the literature and collection of citations was conducted according to the AGREE II criteria and Grading of Recommendations, Assessment, Development and Evaluation methodology. General aspects were addressed in the document, including pathophysiology, genetics, frequency, classification, manifestations and clinical approach, laboratory diagnosis and multidisciplinary evaluation, therapy and supportive measures, follow-up, monitoring, and pregnancy. The CPR document that was developed was intended to be a concise and easy-to-use tool for standardization of care for patients among the healthcare providers that are members of the network or are involved in the care for Pompe disease patients.

**Supplementary Information:**

The online version contains supplementary material available at 10.1186/s13023-024-03373-w.

## Introduction and scope of the paper

This article reports about the development of clinical pathway recommendations (CPRs) for Pompe disease (glycogen storage disease type II) by the lysosomal storage disease subnetwork (LSD-SNW) of MetabERN. MetabERN is a non-profit European Reference Network for Metabolic Diseases consisting of 97 nationally certified centers from 27 European Union (EU) Member States. The network was established by the EU in 2016 to facilitate access to the best available care in the Union and to address the needs of patients affected by inherited metabolic diseases. MetabERN covers different activities, divided into work packages, including the development of recommendations for the diagnosis and management of metabolic diseases.

The Pompe disease CPR document developed by the working group was intended to be a concise and easy-to-use tool for standardization of care for patients among the healthcare providers that are members of the network. The document was based on quality assessment and transparent procedures. The approach used to produce this document was based on literature survey, selection of articles that were deemed valuable and essential for Pompe disease care, quality assessment of the literature, and incorporation of information in a template provided by MetabERN.

In this article the document developed by the working group includes minor adaptations and adjustments according by the Journal’s editorial requirements.

## Methodology

For the development of CPRs a platform was set up on Google Drive and template matrices were made available by the general coordinators of the MetabERN guideline work package.

**Fig. 1:**
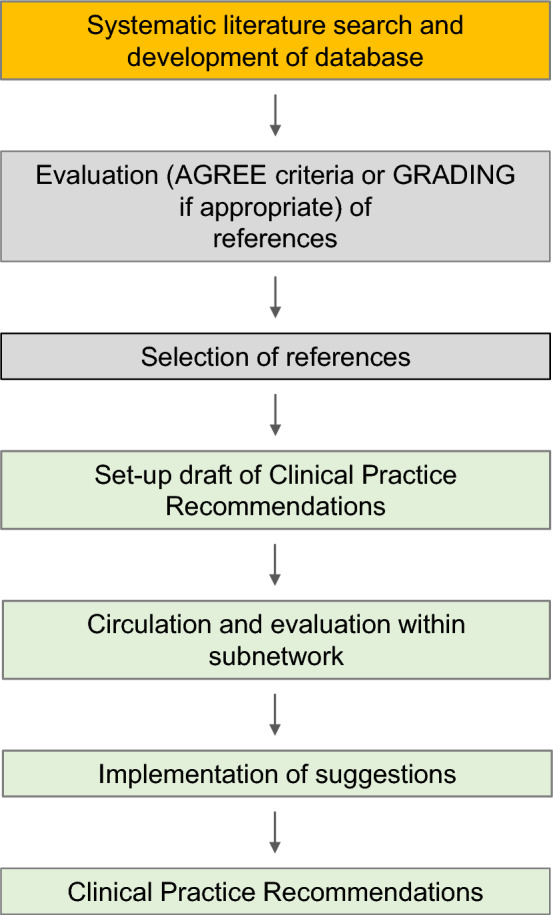
The working strategy for the development of CPRs for Pompe disease. The process was based on a systematic literature search to develop a database, followed by quality assessment of the literature, discussion within the working group, and by the development of the CPR document according to the matrices provided by MetabERN

A working group was selected on a voluntary basis among members of the lysosomal storage disease subnetwork (LSD-SNW). The group was composed by experts from several European countries with specific expertise in the care of Pompe disease, including metabolic physicians, pediatricians, neurologists, child neurologists, endocrinologist, cardiologists, pneumologists, and by patient support group representatives. The work was conducted, according to the workflow indicated by the CPRs platform (Fig. 1), exploiting online resources for exchange of informative materials and correspondence, with periodic reports on advancements and discussions at MetabERN general board conventions or at MetabERN LSD-SNW meetings.

The working strategy was based on a systematic literature search to develop a database, followed by quality assessment of the literature, and by the development of the CPR document according to the matrices provided by MetabERN. An additional file shows the references list evaluated according to the grading system (see Additional file 1).

The literature review was performed in spring 2017 on PubMed, using the following search terms: [“Pompe disease”, or “glycogenosis type II”, or “acid maltase deficiency”, or “acid alpha-glucosidase deficiency”] AND [“guidelines” or “consensus statements”, or “reviews”]. No language or data filters were used. Existing guidelines, consensus clinical protocols, or any single published manuscripts with clear clinical relevance for the CPR development were included in the literature database until the end of guideline development process. Studies published in the timeframe 2000–2016 were included in the literature search. Further revisions of literature and updating of the references were performed at the end of 2021, at the end of 2022, and in May 2024, and again circulated for definitive approval.

For the quality assessment of existing guidelines and consensus the AGREE II were used. Whenever primary data were used (clinical trial, clinical research, basic research) the quality of the paper was assessed by the GRADE system. The level of evidence of individual studies was rated from 4 (lowest) to 1 +  + (highest). The information about selection of published studies and quality assessment is provided as supplementary material S1.

A draft document was circulated among all members of the LSD-SNW (healthcare providers and patients’ association representatives) for evaluation in summer 2019. The document was further discussed at a satellite MetabERN session within the 2019 Annual Meeting of the Society for the Study of Inborn Errors of Metabolism in Rotterdam, the Netherlands, and was revised according to suggestions.

## The clinical practice recommendations for Pompe disease

### Pathophysiology

Pompe disease, or glycogen storage disease type II, is a lysosomal storage disorder and a metabolic myopathy caused by deficiency of lysosomal acid alpha-glucosidase (GAA, also referred to as acid maltase).

GAA hydrolyzes the 1,4 and 1,6 glucosidic bonds of glycogen. This function is required for the breakdown of glycogen into glucose in the lysosomes. Biallelic *GAA* gene pathogenic variants result into absent or deficient activity of the GAA enzyme, which leads to the accumulation of glycogen in the lysosomes of several cell types and tissues, particularly cardiac, skeletal, and smooth muscle cells. In addition to glycogen storage, a typical secondary feature of Pompe disease pathology is the accumulation of autophagic material in muscle fibers [1, 2]. Normal intracellular metabolism becomes disturbed, including mitochondrial function with oxidative stress activation [3, 4], and/or cytoplasmic glycogen metabolism impairment. Disruption of lysosomes by itself, with release of proteolytic enzymes into the cytoplasm, may also play a role in the disease pathophysiology [5].

### Genetics

Pompe disease is inherited in an autosomal recessive manner and is due to biallelic pathogenic variants in the *GAA* gene. The *GAA* gene is localized on chromosome 17 at the 17q25.2–q25.3 locus and contains 20 exons including the 19 coding ones [6, 7].

There is a high allelic heterogeneity/diversity: missense, nonsense, splice-site variants, partial deletions, and insertions have been reported to be causative of the disease. As of December 2020, Pompe disease *GAA* variant database at the www.pompecenter.nl website included 648 disease‐associated variants, 26 variants from newborn screening, and 237 variants with unknown severity [8]. The database is also directly accessible via www.pompevariantdatabase.nl.

The most common pathogenic variant is the intronic mutation c.-32-13T > G (found at the heterozygous state in approximately 80–90% of adult patients and 50% of children) and associated with a slowly progressive course of disease [7]. This splice site mutation results into variable levels of residual activity (up to 20% of normal) and mostly combines in adults with a very severe pathogenic variant on the second allele [8]. Genetic modifiers explaining the broad clinical variability in patients carrying the c.-32-13T > G variant have been identified. For example, the silent, *cis*-acting c.510C > T variant reduces leaky wild type splicing and thereby residual GAA activity [9]. Patients homozygous for the c.-32-13T > G variant rarely express symptoms [10].

Other relatively prevalent mutations show typical ethnical distribution, such as the p.Glu176Argfs*45 (often referred to as c.525del), p.Gly828_Asn882del, and p.Gly309Arg in the Dutch population, p.Arg854* in Africa, p.Asp645Glu in Taiwan, p.Ser529Val, p.Arg672*, p.Arg600Cys in Japan, p.Trp746Cys in China, p.Gly828_Asn882del in Canada [11, 12].

*GAA* variants associated pseudo-deficiency of GAA have been described, for example, the variants p.Gly576Ser and p.Glu689Lys, often present *in cis* [13]. Patients homozygous for these mutations have low levels of GAA activity but do not develop clinical signs of the disease.

Most GAA variants lead to production of some (active or inactive) GAA protein. Patients expressing these variants are called CRIM (Cross Reactive Immune Material) positive. About one third of infantile Pompe disease patients, depending on their genotype, do not express any GAA protein and are defined CRIM negative. For example, the mutation p.Glu176Argfs*45 is a CRIM negative *GAA* gene variant. CRIM negative patients have a higher risk of producing antibodies against recombinant enzymes when treated with enzyme replacement therapy (ERT) [14, 15].

### Frequency

The estimated incidence of Pompe disease has been reported to vary between 1:40,000 and 1:146,000 [7, 16]. Newborn screening programs implemented in some countries have led to reports of figures between 1:8684 and 1:23,596 [17–19]. Recent studies have revealed a similar incidence in some European countries [20, 21].

The incidence rate is higher in specific countries and ethnic groups, such as Taiwan (1 in 17,000) [19] and French Guiana (1 in 2000) [22].

### Classification

Traditionally, different clinical forms of the disease, outlined in Table 1, have been described in the literature depending on age at onset and severity:

1. Infantile-onset Pompe disease.
2. Late-onset Pompe disease or non-classic Pompe disease (childhood, juvenile, adult-onset).

**Table 1:**
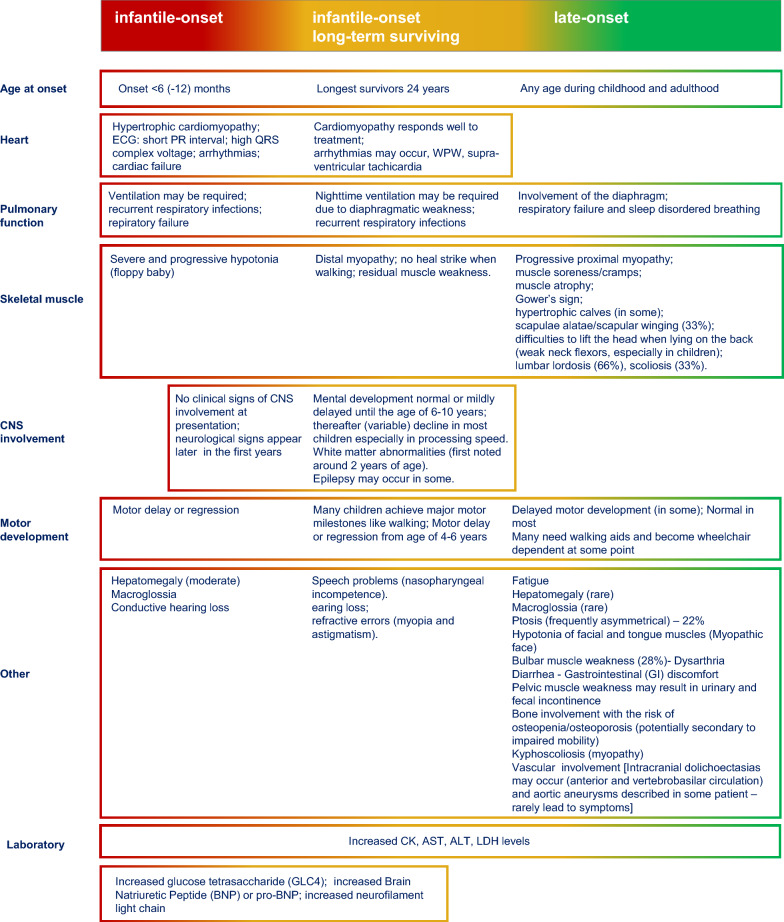
Pompe disease spectrum of manifestations

However, the clinical spectrum of Pompe disease is broad and continuous, and symptoms can manifest at any age from infancy to late adulthood.

Skeletal muscle weakness dominates the clinical picture and affects both respiration (including the diaphragm) and mobility. The course of the condition is variable in older children and adults, but it remains relentlessly progressive, resulting in significant morbidity and often in premature mortality. Respiratory failure is the major cause of death [5].

### Manifestations and clinical approach

1. Infantile-onset Pompe disease (IOPD).

The classic infantile form is the best delineated form of Pompe disease and at the most severe end of the clinical spectrum. The disease may be present at birth or within the first few months of life with hypotonia, feeding difficulties or respiratory problems. A hypertrophic cardiomyopathy is characteristically present and may already develop in utero. Without therapy the disease progresses fast, and patients do not achieve major motor milestones like sitting, standing or walking and die within the first year of life of cardiorespiratory failure.

*Atypical infantile Pompe disease*

Rarely patients with infantile Pompe disease present later (beyond 6 months of age). This atypical form of infantile Pompe disease should be suspected in infants that present within the first two years of life with generalized hypotonia, cardiac hypertrophy, mild liver enlargement, recurrent respiratory infections (due to cardiac disease and hypotonia/weakness of respiratory muscles), macroglossia. Cardiac hypertrophy is mostly less prominent than in the classic form. Development of motor milestones is delayed. Some of these children achieve the ability to sit or stand without therapy.

Together the classic infantile form and the atypical infantile form are frequently named infantile onset Pompe disease. Since patients with the atypical form have a better prognosis, it is important to make the differentiation.

ERT has changed the prospects of patients with infantile Pompe disease dramatically. Overall survival has increased, particularly in children with high-dosage treatment regimens (see also section “Therapy”). Many children learn to walk. However, children are not cured. A new phenotype has emerged in long-term surviving patients.

2. Late-onset Pompe disease (LOPD)

The phenotype of late-onset Pompe disease is extremely broad and is generally associated with slower disease progression [23, 24]. Patients may present at any age, but mostly after the age of 1 year during childhood or adulthood. They usually present with proximal (limb girdle) myopathy leading to progressive motor disability (more closely related to disease duration than to the age of the patient), with waddling gait, mostly without cardiac involvement. Respiratory muscle involvement may occur early in the course of the disease. Due to the involvement of diaphragm, pulmonary function in supine position may be more affected than in upright position. Respiratory involvement can be accompanied by headache, somnolence, and/or dyspnea. Respiratory and motor involvement do not necessarily have to progress at the same rate. Rarely patients present with respiratory failure.

Smooth muscles may be involved with, as an example, dolichoectasia of cerebral vessels, but only a very few cases have been described in which an aneurysm has led to intracerebral hemorrhage.

Mild myopathic features, creatine kinase (CK) levels < 1000 U/L in adults and up to 2500 IU/l in childhood onset patients and proximal limb girdle weakness and/or axial muscle weakness with or without reduced pulmonary function, in particular when in supine position should be considered as red flags for LOPD patients [25].

### Diagnosis

#### Newborn screening

Newborn screening (NBS) for Pompe disease is possible by measuring GAA activity in dried blood spots with different methods (tandem-mass spectrometry, fluorometry, microfluidics) [17–21, 26]. Newborn screening is essential for timely identification and treatment of patients with the infantile-onset forms of the disease.

Targeted next generation sequencing (NGS) could provide additional information and confirmation of the diagnosis for people identified by biochemical screening [27].

However, some limitations of the newborn screening should be considered. First, the assay in dried blood spots is only a screening test and is not sufficient for definitive diagnosis. Second, the NBS screening in its current form cannot discern IOPD from LOPD. LOPD patients are thereby patients in waiting requiring long term follow-up and monitoring which may create uncertainty and a psychological burden for families [28, 29].

NBS programs are already active in several countries (for example, in the US, Taiwan, Japan, some Italian regions) [19, 20, 27]. Pompe disease was added to the US Recommended Universal Screening Panel (RUSP) in 2015 [30].

#### GAA enzyme assay

A GAA enzyme assay in dried blood spot assay can be used as a first line test. However, this test is not sufficient for a definitive diagnosis. The diagnosis of Pompe disease should be confirmed by GAA enzyme assay in at least one of the following: peripheral leukocytes/lymphocytes, cultured fibroblasts from skin biopsy, muscle biopsy. Common biochemical assays are based on the use of the artificial fluorogenic substrate 4-methylumbelliferyl-α-D glucopyranoside (4MUG) [13]. The discovery that acarbose is a selective inhibitor of maltase glucoamylase allows acid alpha-glucosidase to be selectively assayed in white blood cells and dried blood spots [31].

The possibility of GAA pseudo deficiency should be considered for the interpretation of the GAA biochemical assay (see section “Genetics”) [32]. The use of glycogen as natural substrate enhances the resolution between affected and unaffected; however, the GAA2 pseudo deficiency that occurs in the Caucasian population, can be excluded using 4MUG rather than glycogen [13].

GAA residual enzyme activity in general correlates with phenotype severity, with the lowest activities (< 1%) found in classic infantile patients, and activities from 2 to 40% in late-onset attenuated phenotypes [5].

#### Molecular analysis of the GAA gene

The molecular analysis of the GAA gene should follow the enzyme assay. This test is useful for further diagnostic confirmation and is necessary for the genetic counseling. Variant classification should follow the American College of Medical Genetics and genomics and Association for Molecular Pathology (ACMG-AMP) system of variant classification which includes 5 classes: benign, likely benign, variant of unknown significance (VUS), likely pathogenic, and pathogenic (class 5 providing ultimate proof of pathogenicity, see for guidance www.pompevariantdatabase.nl). In addition, considering current knowledge about genotype–phenotype correlations, molecular analysis of the GAA gene may provide information about prognosis [7, 33].

The combination of a pathological GAA assay and a genetic confirmation represents the gold standard for Pompe disease diagnosis. This approach is supported by expert consensus statements published in the literature [34, 35], with a moderate-high level of evidence.

Recently, NGS approaches have been exploited in cohorts of patients with skeletal muscle diseases and limb-girdle muscle dystrophies and have allowed for identification of misdiagnosed Pompe disease patients [36].

When clinical suspicion is strong and standard procedures are insufficient, additional molecular methods may be required to validate the diagnosis of Pompe disease, such as a generic splice assay (consisting of exon-flanking RT-PCR and exon-internal RT-qPCR), MLPA, minigene analysis, SNP array analysis, and targeted Sanger sequencing [37].

### Complementary laboratory tests

Routine blood chemistry usually shows increased serum levels of AST, ALT, CK, LDH.

A rapid and simple complementary test to identify affected subjects is based on the detection in peripheral blood smears of PAS-positive vacuoles in lymphocytes [38].

In patients with infantile Pompe disease it is important to test for cross-reacting immunologic material (CRIM) status of patients through a Western blot analysis or DNA analysis. Studies in multiple cohorts of patients support the concept that CRIM status may be informative as a prognostic factor and as a predictive element of response to ERT since CRIM negative patients are more likely to develop antibodies against GAA [14, 15].

Analysis of some biomarkers, when available, may be performed, for example the brain natriuretic peptide (BNP) or pro-BNP, reflecting improved cardiac function [39]; the urinary glucose tetrasaccharide (Glc4) [40]; specific skeletal muscle-enriched microRNAs [41, 42]; neurofilament light chain [43, 44].

### Multidisciplinary evaluations at diagnosis

Clinical multidisciplinary evaluations at the time of diagnosis or as an initial assessment should include:

For infants with classical IOPD (see also Table 2):

**Table 2:** Infantile onset Pompe disease—follow-up exams and investigations

	Basic investigations																
Last evaluated (Date)								Cardiology			Neuromuscular evaluation			Radiology			
	History and status	Vital parameters, RR, weight, height, BMI, abdominal circumference	Enzyme activity	Genetics	Metabolic Laboratory	Antibody titer	Routine laboratory	Chest X-ray	ECG, echocardiogram	24 h ambulatory ECG	MMT-MRC(1)	6-MWT(1)	timed test	Lung MRI or chest CT or B-mode ultrasound diaphragm	Skeletal muscle MRI	Cardiac MRI	Brain MRI
First presentation	X	X	X	X	X	X	X	X	X	X	X	X		X	X	X	
3 Mo	X	X								X		X	X				
6 Mo	X	X				X	X				X	X	X				
9 Mo	X	X								X		X	X				
12 Mo	X	X			X		X	X(2)			X	X	X	X(3)			
Annual investigations	X	X				X	X	X				X	X	X			
Each 2nd year																	X(1)
Each 3rd year										X					X(3)		
Each 5th year																	
If needed								X	X	X				X	X		

	Basic investigations											Nice to have		
Last evaluated (Date)	Gastroenterology			Pneumology										
	Videofluoroscopic swallowing assessment	Liver ultrasound	Nutritional status	Pulse oxymetry	FVC sitting/ supine (1)	MIP/ MEP (1)	Polysomnography	Hearing tests including otacoustic emission tympanometry	Ophthalmological evaluation	Language, speech, and oromotor function	DEXA scan	Cognitive assessment/ psych	Skeletal x-ray	
First presentation	X	X	X	X	X	X	X	X	X	X	X	X	X	X
3 Mo				X										
6 Mo			X	X										
9 Mo				X										
12 Mo	X(3)			X	X		X	X				X		
Annual investigations	X(3)				X	X		X	X	X	X			
Each 2nd year		X										X		
Each 3rd year														
Each 5th year												X	X (4)	
If needed	X			X									X	

(1) Compatibly with patients’ clinical conditions, age and participation

(2) More frequently in the presence of HCMP

(3) More frequent if required, depending on the patient's condition

(4) More frequent if required (eg spine deformities)

*General*

- Physical examination.
- Growth parameters.

*Neuromuscular evaluation*

- Motor and functional assessments compatibly with patients’ clinical conditions, age and participation.

*Neurodevelopmental assessment* (specifically in infantile patients)

- Neuropsychological evaluation and developmental tests (as appropriate for age).

*Cardiology*

- Chest X-ray.
- ECG.
- Echocardiogram—cardiac ultrasound scan (to evaluate hypertrophic cardiomyopathy).
- 24-h ECG.

*Pneumology and respiratory function tests*

- Pulse oximetry.
- Polysomnography.
- Assessment of need for ventilatory support by home ventilation experts (if applicable).

*Gastrointestinal and nutritional evaluation*

- Video fluoroscopic swallowing assessment and evaluation for gastro-esophageal reflux to guide management of feeding (oral/gavage feeding).
- Liver ultrasound scan.
- Nutritional status and nutrient (protein) intake.

*Radiology and imaging*

- Chest radiography in infants will show an enlarged heart in infants and possible skeletal/spine deformities.

*Auditory function*

- Hearing tests including otoacoustic emissions, tympanometry, and brain auditory evoked potentials (ABR/BAEP).

*Ophthalmological evaluation*

- Visual acuity test. Myopia frequently occurs in patients with the classic infantile form.
- Orthoptic evaluation.

*Language, speech, and oromotor function*

- Assessment batteries for speech intelligibility, disordered articulation, and hypernasality.

For patients with LOPD (see also Table 3)

**Table 3:** Late onset Pompe disease: follow-up exams and investigations

Last evaluated (Date)	Basic investigations																	
								Cardiology			Neuromuscular evaluation					Radiology		
	history and status	Vital parameters, RR, weight, height, BMI, abdominal circumference	Enzyme activity	Genetics	Metabolic Laboratory	Antibody titer	Routine laboratory	Chest X-ray	ECG, echocardiogram	24 h ambulatory ECG	MMT-MRC	6-MWT	Hand-held dynamometry	Timed test	Patient-reported outcome measures	Lung MRI or chest CT or B-mode ultrasound diaphragm	Skeletal muscle MRI	Cardiac MRI
First presentation	X	X	X	X	X	X	X	X	X	X	X	X	X	X	X	X	X	X
3 Mo																		
6 Mo	X	X				X	X				X	X	X	X	X			
9 Mo																		
12 Mo	X	X			X		X	X(1)			X	X	X	X	X	X(1)		
annual investigations		X			X	X	X				X	X	X	X	X			
each 2nd year																		
each 3rd year										X							X(2)	
each 5th year																		
if needed								X	X	X						X	X	

Last evaluated (Date)	Basic investigations									Nice to have		
	Gastroenterology			Pneumology								
	Videofluoroscopic swallowing assessment	Liver ultrasound	Nutritional status	Pulse oxymetry	FVC sitting/supine	MIP/MEP	Polysomnography	Hearing tests including otacoustic emission tympanometry	Language, speech, and oromotor function	DEXA scan	Cognitive assessment/psych	Needle elcetromyography (EP)
First presentation	X	X	X	X	X	X	X	X	X	X	X	X
3 Mo												
6 Mo			X									
9 Mo												
12 Mo	X(1)						X	X	X		X	
annual investigations	X(1)			X(1)	X		X	X	X			
each 2nd year		X									X	
each 3rd year												
each 5th year											X	
if needed	X			X								

(1) More frequent depending on the patient's condition

(2) More frequent if required (eg spine deformities)

*General*

- Physical examination.
- Growth parameters.

*Neuromuscular evaluation*

- Motor and functional assessments. As LOPD patients may present at any age, depending on their age and level of participation: 6-min walking test (6-MWT) (from the age of 2), Muscular force by Medical Research Council (MMT-MRC) (from the age of 5), timed tests, hand-held dynamometry (from the age of ten), patient-reported outcome measures [39].
- Needle electromyography (EMG). EMG and peripheral nerve conduction studies are optional and may be considered at diagnosis as a supportive element.
- Muscle biopsy (not needed when other biochemical tests are conclusive for the diagnosis).

*Cardiology*

- ECG.
- Echocardiogram—cardiac ultrasound scan.
- Twenty four-hour ambulatory ECG.

*Pneumology and respiratory function tests*

- Pulse oximetry.
- Spirometry: forced vital capacity (FVC) sitting; FVC supine; Maximun Inspiratory Pressure and Maximum Expiratory Pressure (MIP/MEP) (from the age of 6).
- Polysomnography.
- Assessment of need for ventilatory support by home ventilation experts (if applicable).

*Gastrointestinal and nutritional evaluation*

- Video fluoroscopic swallowing assessment and evaluation for gastro-esophageal reflux to guide management of feeding (oral/gavage feeding).
- Liver ultrasound scan.
- Nutritional status and nutrient (protein) intake.

*Auditory function*

- Hearing tests including otoacoustic emissions, tympanometry, and auditory evoked potentials (ABR/BAEP).

*Language, speech, and oromotor function*

- Assessment batteries for speech intelligibility, disordered articulation, and hypernasality.

*Ophthalmological evaluation*

- Visual acuity test.
- Orthoptic evaluation.

*Others*

- Psychological evaluation.
- Quality of life scales.

*Radiology and imaging*

- Dual-energy X-ray absorptiometry (DEXA) scan (to screen for osteopenia/osteoporosis) in adult patients.
- Skeletal X-ray in the presence of skeletal dysmorphisms.

*Additional evaluations for both IOPD and LOPD*

There are several additional imaging techniques that may be available in centers with expertise in the management of Pompe disease and may be advisable to perform both in IOPD and LOPD patients. Even though these tests may be of help in the assessment and evaluation of patient clinical conditions, they require specific experience and skills, and should not be considered as routine or indispensable procedures. These include:

- B-mode ultrasound to assess diaphragm thickness and search for diaphragm paralysis and computed tomography (CT) scan for evaluation of lungs and diaphragm thickness.
- Magnetic Resonance Imaging (MRI). If compatible with patients’ conditions (the supine position might be associated with aggravated respiratory failure) and with the need for sedation, brain MRI may provide useful information on:
- Respiratory muscles, position, and thickness of the diaphragm [45].
- Skeletal muscle trophism and fatty degeneration. Whole-body MRI protocols are more inclusive than standard MRI protocols focusing on specific anatomical regions (e.g., paraspinal muscles, tongues, pelvis, thigh), enabling evaluation of relevant muscle groups beyond the pelvis and proximal lower extremities [46].
- Brain involvement (in infants compatibly with patient conditions). Recent evidence indicates that classic infantile patients may show white matter abnormalities [47]. So far, they have not been encountered in patients with the atypical infantile form. As these manifestations are not present until later in life, a brain MRI may not be required at the first assessment.

In LOPD patients cerebrovascular manifestations (e.g., aneurysms, vertebrobasilar dolichoectasia, dilatative arteriopathy) have been reported [48].

For most of the basic evaluations there is sufficient support and good quality evidence in the selected literature. The level of agreement on their importance for an accurate assessment of patients’ status is high.

For additional evaluations the indications are somehow less stringent, probably because of a lower number of studies or because some aspects of the disease have been identified only in relatively recent years (for example central nervous system involvement in IOPD patients); thus, the level of evidence in the literature can be assessed as moderate-high.

### Differential diagnosis

Depending on the clinical form, differential diagnosis with other disease entities should be considered (Table 4).

**Table 4:** Differential diagnosis

*Late-onset patients*	
Muscular dystrophies	Becker muscular dystrophy Limb-girdle muscular dystrophies Scapulo-peroneal muscular atrophy Rigid spine syndrome
Genetic metabolic Diseases	Glycogen storage diseases (debrancher deficiency, branching enzyme deficiency, myophosphorylase deficiency, phosphofructokinase deficiency) Danon disease Mitochondrial disorders (respiratory chain disorders, beta-oxidation defects)
Inflammatory myopathies	Polymyositis

*Infantile-onset patients and juveniles*	
Spinal muscular atrophy	Acute Werdnig-Hoffman disease
Muscular dystrophies	Congenital muscular dystrophies (Duchenne/Becker, Emery Dreyfuss, limb-girdle)
Congenital myopathies	Nemaline myopathy, fiber type disproportion, central core myopathy
Inborn metabolic Diseases	Glycogen storage diseases Mitochondrial disorders Peroxisomal disorders Congenital defects of glycosylation (CDG) Very long-chain acyl-CoA dehydrogenase (VLCAD) deficiency
Congenital cardiac Diseases	Idiopathic hypertrophic cardiomyopathy Myocarditis Endocardial fibroelastosis
Lysosomal storage Diseases	Danon disease
Other	Hypothyroidism

### Therapy

#### Therapeutic goals

The therapeutic goals in Pompe disease are:

Infants

1. Improving survival.
2. Improving or normalizing cardiorespiratory function.
3. Improving or preserving normal motor skill acquisitions.
4. Normalizing growth.
5. Preventing need for ventilator support.

Late-onset patients

1. Reducing or stabilizing musculoskeletal damage in symptomatic patients.
2. Improving or stabilizing respiratory function.
3. Improving the nutritional state of the patient.
4. Preventing skeletal dysmorphisms (particularly kyphoscoliosis).
5. Improving quality of life.

*Enzyme replacement therapy (ERT) with recombinant human GAA (rhGAA).* (Table 5).

**Table 5:** Enzyme replacement therapy for Pompe disease

Recombinant human GAA formulation	Licensed dose	Note	Efficacy
Alglucosidase alfa	20 mg/kg/eow	Dose may be increased up to 40 mg/kg/eow or 40 mg/kg/w in patients with classic infantile and in late onset patients showing a suboptimal response, plateau, or clinical decline	Approved in 2006. Since then, a large number of studies on the efficacy of alglucosidase alfa has been published in infantile-onset and in late-onset Pompe disease patients Infantile-onset patients: Long-term alglucosidase alfa treatment substantially improves cardiomyopathy, markedly extends survival and ventilation-free survival Iate-onset patients: alglucosidase alfa treatment improves motor (6-min-walk test, 6-MWT) and respiratory function (forced vital capacity, FVC). Little or no difference in quality-of-life physical component score
Avalglucosidase alfa	20 mg/kg/eow		Approved in 2021. Still limited evidence based on Avalglucosidase alfa versus alglucosidase alfa studies Avalglucosidase alfa probably improves 6-MWT compared to alglucosidase alfa. Avalglucosidase alfa probably makes little or no difference to % predicted FVC compared to alglucosidase alfa For infantile-onset patients who experience lack of improvement or insufficient response a dose increase to 40 mg/kg/eow may be considered
Cipaglucosidase	20 mg/kg/eow	Approved in association with Miglustat	Compared to alglucosidase alfa plus placebo, cipaglucosidase alfa plus miglustat probably improves % predicted FVC compared to alglucosidase alfa plus placebo Compared to alglucosidase alfa plus placebo, Cipaglucosidase alfa plus miglustat may make little or no difference to: 6-MWT distance; quality of life scores for physical function and fatigue

Sources of data on efficacy:

Dalmia S, Sharma R, Ramaswami U, Hughes D, Jahnke N, Cole D, Smith S, Remmington T. Enzyme replacement therapy for late-onset Pompe disease. Cochrane Database Syst Rev. 2023 Dec 12;12(12)

Chen M, Zhang L, Quan S. Enzyme replacement therapy for infantile-onset Pompe disease. Cochrane Database Syst Rev. 2017 Nov 20;11(11)

The rhGAA preparation Alglucosidase alfa was approved for the treatment of Pompe disease in 2006 and most of the experience gathered on the efficacy of ERT in Pompe disease has been obtained with this preparation. Alglucosidase alfa has been shown to be effective in improving or stabilizing the disease course both in infantile-onset and in late-onset patients [49–54]. The level of evidence on the effects of ERT both in infantile-onset and late-onset Pompe disease patients is based on long term, high-quality clinical studies in large numbers of patients. The level of evidence is high.

Two other rhGAA preparations, both enriched in their mannose-6-phosphate content and with improved muscle-targeting properties, were granted approval in recent years [55, 56]. Avalglucosidase alfa was approved by Food and Drug Administration (FDA) in 2021 and by European Medicines Agency (EMA) in 2022 for the treatment of late-onset Pompe disease.

Cipaglucosidase received approval by EMA in 2023, also for the treatment of late-onset patients and in the US for ERT experienced patients to be switched from Myozyme to Cipalglucosidase. [57] Each of these preparations were evaluated in large phase 3 studies.

##### Dose

The licensed dose of Alglucosidase alfa is 20 mg/kg body weight, every other week, by intravenous infusion. Published studies have shown that higher rhGAA doses (from 20 mg/kg every other week to 40 mg/kg/week) may be clinically appropriate and safe in infantile-onset patients, improving gross motor outcomes, pulmonary function measures, and biochemical markers [50–53]. Further, a high-dose regimen of 40 mg/kg week showed a better effect on survival and also on walking ability than the recommend dose in classic infantile patients [58–60]. High-dose rhGAA may also be a treatment option for late-onset Pompe disease patients showing a suboptimal response, plateau, or clinical decline at the standard dose, in the absence of infusion-associated reactions and clinically significant anti-rhGAA neutralizing antibody titers, but further studies are needed to demonstrate this effect.

While the efficacy of the licensed dose is based on long term experience in large numbers of patients, the evidence supporting the use the higher doses in infantile-onset patients has been obtained in a limited number of clinical studies and the level of evidence is moderate-high.

Avalglucosidase alfa licensed dose is 20 mg/kg body weight/every other week [61]. For infantile-onset patients who experience lack of improvement or insufficient response in cardiac, respiratory, and/or motor function while receiving 20 mg/kg, a dose increase to 40 mg/kg every other week may be considered.

The recommended dosage of Cipaglucosidase alfa is 20 mg/kg every 2 weeks [57] and has been tested in combination with miglustat [56].

For the second-generation recombinant enzymes (Avalglucosidase alfa, Cipaglucosidase) the efficacy has been assessed in a limited number of studies [55, 57, 62–64]. These studies suggest significant improvements or stabilization of some clinical manifestations [54].

##### Indications

Criteria for start and stop treatment in infantile-onset Pompe patients is under evaluation by a European expert panel (EPoC, European Pompe disease Consortium). In these patients treatment should be started immediately after diagnosis, without delay. Early initiation of ERT in infantile-onset patients contributes to a better physical and developmental outcome [60]. Timely start of ERT is associated with preservation of FVC in late-onset patients with better respiratory function and positive effects on walking ability at the time of treatment initiation, but not all patients respond equally well [65].

While the level of evidence for starting early ERT in infantile onset patients is high, evidence for exclusion or stopping therapy criteria is low and is not sufficiently supported by literature. When patients present with extremely severe clinical manifestations, are already invasively ventilated and without any residual respiratory and skeletal muscle function, and no beneficial effects of ERT are expected, it may be reasonable to refrain from starting treatment, or to stop treatment, after extensive discussion with parents. However, the authors are aware that protocols for the start of ERT in classic infantile patients may differ between countries.

Consensus on criteria to start, switch and stop therapy in late-onset patients has been reached by the same expert panel [66]. Specifically, to start ERT a patients should have an established diagnosis of Pompe disease, should present with clinical and supportive paraclinical signs of the disease, should have functionally relevant residual skeletal and respiratory function, should not have another advanced stage life-threatening disease, should be committed to continue treatment. The absence of residual skeletal or respiratory function, the presence of another advanced stage life-threatening disease, an insufficient commitment of patients to treatment may represent reasons for not to recommend the start of ERT. Stopping treatment should be considered for unmanageable severe infusion-associated reactions, high neutralizing antibody titers, lack of any effect of treatment, patient wish, another advanced stage life-threatening disease represent criteria to consider stopping ERT.

Switching to a second-generation ERT can be considered if there is no indication of skeletal muscle and/or respiratory function stabilization or improvements after at least a year on first-generation recombinant alpha-glucosidase, or if the patient suffers from severe infusion-associated reactions that cannot be adequately managed.

The level of agreement is based on published consensus criteria and is considered high.

ERT should be prescribed (and its effects monitored) by centers with specific expertise in the treatment of Pompe disease and/or other lysosomal diseases. rhGAA is approved for hospital administration in different countries and enzyme infusion can take 3–6 h. Home therapy could ameliorate the patient quality of life, although there is the potential for severe infusion reactions and life-threatening anaphylaxis in patients receiving ERT [67, 68].

##### Immune tolerance induction

Immune-modulating protocols have been proposed to counteract neutralizing antibodies to rhGAA in infantile onset forms, mainly in CRIM-negative patients. Recent protocols involve variable combinations of rituximab, methotrexate, bortezomib, rapamycin [15, 69–71], plasma-exchange [72] and support with gamma globulins [73].

Since prophylactic induction of immune tolerance must begin prior to the first rhGAA infusion, it is important to rapidly determine CRIM status. However, this should not delay the start of the ERT. Therefore, it may be advisable that infantile-onset patients with unknown CRIM status are treated as if they were CRIM negative [39]. It should also be noted that 30% of CRIM positive patients develop high sustained antibodies. Although common practice between centers may vary, for the most up-to-date protocols for ERT-naïve and ERT-experienced patients with high sustained antibody titers we refer to Banugaria et al. [15] and Desai et al. [74].

Protocols to desensitize PD patients with infusion-associated reactions due to ERT hypersensitivity have been published [75, 76] but are not generally applied/recommended.

#### Other therapeutic approaches under development

Beta-2 adrenergic agonist, such as albuterol, has been investigated and tested in clinical trials as an add-on therapy which may enhance the lysosomal uptake of rhGAA [77–79].

In vivo and *ex-vivo* gene therapy approaches are under clinical development [80, 81].

Substrate reduction therapy based on oral administration of small-molecule muscle glycogen synthase (GYS1) inhibitors is currently under investigation [82].

#### Diet

Recommended diet composition:

25–30% proteins;

30–35% carbohydrate;

35–40% lipids.

It is important to ensure suitable calorie and protein intakes, and to avoid catabolism. It has been advised that adult Pompe patients should consume 1.2–1.4 g/kg protein per day, which is above the intake recommended for the general population (0.8–1.0 g/kg) [35]. The rationale for a high-protein diet is to counteract muscle protein depletion by supplying increased amino acid substrates for protein synthesis.

Supplementation with L-alanine has been proposed as an alternative way to reduce muscle protein turnover and thus possibly improve muscle function [83].

Evaluation of personalized diets is recommended as Pompe patients tend to get more overweight than others.

#### Other supportive therapies

In addition to ERT, palliative, rehabilitative, supportive, and surgical therapies are needed to manage pulmonary, cardiac, musculoskeletal, neurological, gastrointestinal and psychological issues. Speech therapy should be considered in infants.

These therapies should be performed in centers with specific expertise in the management of Pompe disease or neuromuscular disorders in general and should be based on multidisciplinary evaluations.

##### Cardiac involvement

Therapies for cardiac failure, may be required, mostly in infantile-onset patients.

Infantile-onset patients with cardiomyopathy should initially avoid digoxin or inotropes since they can worsen left ventricular outflow status [84]. Drug therapy with Angiotensin Converting Enzyme inhibitors, calcium antagonists and beta-blockers can be indicated, but these medications should be used with caution and only by a pediatric cardiologist experienced in treating pediatric patients with heart failure [39].

##### Physical therapy and exercise

Active muscle strengthening exercises, aerobic exercise therapy and home exercise program are beneficial [85, 86], depending on patients’ conditions. Passive mobilization and physiotherapy may help prevent joint contractures and deformities. Rehabilitation programs should be defined by an experienced team. Splints may help to counteract shortening of the Achilles tendons/clubfoot.

##### Respiratory therapy and ventilation

Oxygen supplementation and/or non-invasive positive pressure ventilation should be prescribed based on underlying ventilatory abnormalities such as hypoxemia, obstructive sleep apnea, and hypoventilation. Treatment modality and need for mechanical ventilation should be based on careful evaluation of respiratory function by home ventilation experts.

In Pompe disease the diaphragm is involved leading to lower pulmonary function in supine position than in upright position and patients may need nighttime ventilation. Patients with an FVC < 40% should be brought to the attention of a home ventilation team [87, 88].

Procedures to facilitate clearance of airway secretions should be routinely performed [89].

##### Vaccinations

Routine immunizations, including pneumococcal vaccination, should be used. Vaccination for influenza may be advisable for patients and other household contacts [90]. Respiratory syncytial virus (RSV) prophylaxis (palivizumab) is indicated in the first two years of life [91].

##### Management of feeding

The management of feeding (oral or gavage) should be guided by the video fluoroscopic swallowing assessment and evaluation for gastro-esophageal reflux [90].

Nissen fundoplication can reduce the risk of aspiration in those with severe gastro-esophageal reflux [35].

### Emergency

#### Acute respiratory failure (ARF)

Acute respiratory failure is the most frequent cause of death independent of the rate of progression of disease. It is more commonly a consequence of respiratory tract infection in patients with known ventilation defect. When hospitalization is needed, the preferable location is a respiratory intensive care unit (RICU).

Management of ARF includes standard measures including the following:

- Mechanical ventilation, preferably Non-Invasive Positive Pressure Ventilation (NPPV) to assist inspiratory muscles.
- It should be noted that most patients have lower FVC in supine position due to poor diaphragmatic function; therefore, they should not lay totally flat, but be positioned in a (slightly) upright position.
- Optimal oxygen supplement when required (maintain saturation between 92 and 94% and monitor PaCO_2_ and pH).
- Airway secretion clearance through cough assist devices and other physiotherapeutic techniques.
- Endotracheal intubation, only in severe cases.
- Nutritional therapy to reduce aspiration risk.
- Physiotherapy to reduce joint problems.
- Aggressive infection treatment.

ERT should not be suspended [90].

#### Adverse reactions to ERT

Patients on ERT should be monitored for the possible occurrence of adverse reactions during infusions or in the hours after infusions. Fever, chills, erythema, drop of oxygen saturation and others immune and anaphylactic reactions might occur. Management of adverse effect includes the following measures, according to the standard protocols for infusion-associated reactions:

- interruption of the infusion and restart at a reduced infusion rate.
- H1-antihistamines, corticosteroids and epinephrine, which should be readily available (already prepared in the syringe) when administering infusions [5].

As stated above some desensitisation protocols for the management of infusion-associated reactions due to ERT hypersensitivity have been published [75, 76] but there is limited evidence in the literature supporting their efficacy.

### Follow-up and monitoring of patients

Following diagnosis and baseline full clinical assessment patients should undergo periodic evaluation and examinations to explore heart, respiratory and muscle function (Tables 2 and 3). It is advisable that follow-up programs are individualized and adjusted to the stage of disease.

*General evaluation*

- Growth parameters should be evaluated at regular intervals in infants and children (every 3–6 months, depending on age and clinical forms).

*Musculoskeletal and functional tests*

- Motor and functional assessments should be performed every 3–6 months for children under the age of five, every 6–12 months for older children and adults.

*Neuromuscular evaluation*

A minimum set of tests should be available at the follow-up center [39]:

- Muscular force by Medical Research Council (MMT-MRC) scale (from the age of 5).
- Six-minute walking test and timed tests in ambulant patients (from the age of 2).
- Timed tests.
- Hand-held dynamometry (from the age of 10).
- Fatigue by the fatigue intensity scale (FSS).
- Patient-reported outcome measures.

Motor function in infants can be assessed by the Alberta Infant Motor Scale (AIMS), by the PEDI Pompe test (Pediatric Evaluation of Disability Inventory), or also by Bayley scale depending on patient’s age.

Quantitative muscle MRI can be performed in late-onset patients in addition to annual investigations.

Evaluation procedures should be performed by experienced rehabilitation physicians/physical therapists.

#### Cognitive assessment

Developmental and cognitive assessment in infantile patients at diagnosis and every 12–24 months, using standardized tests appropriate for age.

#### Cardiology

ECG, Echocardiogram, 24 h-Holter. ECG and Echocardiogram should be performed at diagnosis, and at regular intervals (every 12 months or more frequently, depending on patients’ conditions, in the presence of cardiomyopathy).

#### Respiratory function

- Pulmonary function (FVC sitting, supine) should be evaluated in both sitting and supine position at least once a year or more frequently depending on patients’ conditions.
- Pulse oximetry with capnography and/or gas exchange monitored every 6 months in patients with abnormal FVC or if manifestations of intercurrent infections or accelerated worsening become evident.
- Chest radiographs should be performed whenever necessary based on patients’ conditions or in case of intercurrent infections.
- Polysomnography and/or oxycapnography should be performed every 12 months.

Evaluation procedures should be performed by an experienced team of ventilation experts.

#### Gastrointestinal function

Assessment and evaluation for gastroesophageal reflux should be performed at the diagnosis and every 3 years or more frequently in the presence of clinical manifestations such as swallowing difficulties, choking respiratory problems and repeated infections. In the absence of clinical problems, video fluoroscopic exam should be considered every 3 years. Oral feeding in infantile patients with Pompe disease should be stopped if there signs of aspiration on video fluoroscopic exam and be restarted when these signs have disappeared.

Nutritional status should be assessed every 6–12 months in children, 12–24 months in adults.

#### Auditory function

Hearing tests should be performed at the diagnosis in infants and every 12 months.

#### Ophthalmological evaluation

- Visual acuity test.
- Orthoptic evaluation.

#### Bone density

DEXA scans and radiographs should be performed at diagnosis and every 5 years or more frequently in the presence of clinical manifestations indicating progression of bone involvement/fractures.

#### Anesthesiologic evaluation

General anesthesia should be limited to a minimum, particularly in young infants and should only be carried out by anesthetists with experience in managing general anesthesia in children with heart disease [35].

#### Antibody status

It is a complementary study. Regular determination of antibody status in patients on ERT, at baseline and after ERT is useful to select patients that could benefit of secondary immunomodulation. Ideally, antibody measurement should be conducted at baseline and then at regular intervals (every 6–12 months), although this may depend upon the patient’s clinical status and may be required in case of unexpected worsening of disease course.

#### Quality of life (QoL)

Pompe disease affects patient quality of life (QoL). Reliable approaches to test QoL and participation are Short Form 36 (SF-36) and the Rotterdam Handicap Scale (RHS) [92].

#### Behavior

Use of standardized behavioral checklists to better characterize the behavioral, emotional and social functioning of children and adolescents with Pompe disease over time. These measures are useful screening tools for clinicians to identify potential behavioral and emotional problems in children with Pompe disease in a timely manner and to refer them for further evaluation and treatment [93].

#### Other

When possible, downloadable applications on mobile phone (for example in Italy the AlGkit) may be of help in the clinical management of patients with Pompe disease, to allow continuous remote monitoring of patients by healthcare providers. Such tools can be especially useful in situations such as the COVID19 pandemics to manage related difficulties (reports on patients who have suspended ERT, difficulties in contacting doctors, etc.) [94].

#### Biochemical markers

- Routine biochemistry.
- Serum CK, CK-MB, AST and ALT.
- BNP or pro-BNP (in patients with cardiac involvement).

When possible, depending on the availability of tests at the follow-up center:

- Urinary Glc4 or Hex4.
- neurofilament light chain (in infantile-onset patients).
- muscle specific microRNAs.

### Interaction with patient associations

It is important to inform patients and families about the existence of patients’ associations. This contributes to management of the disease by promoting cooperation, exchange, dialogue and even support between patients, patients' associations and caregivers.

### Pregnancy

While fertility is not affected, pregnancy may worsen symptoms, or cause initial symptoms to arise. Complications with pregnancy, delivery or birth were not higher, except for an increase in the rate of stillbirths (3.8% compared to the national average of 0.2–0.7%) [95–97]. Pregnancy should be carried out in a referral center with the support of a neonatal ward.

Pregnancy induces a host of adaptive changes that may worsen signs or cause arising of initial symptoms of Pompe disease in the mother, putting at risk both the mother and the fetus.

Although there are no adequate and well-controlled studies in pregnant women about the ERT effects, it has been reported that cessation of ERT in early pregnancy may result in deterioration of maternal symptoms and emergence of allergic reactions on restarting ERT [95, 96].

In animal reproduction studies, no effects on embryo-fetal development were observed in mice or rabbits given daily administration of alglucosidase alfa at the recommended human bi-weekly dose during the period of organogenesis. Although the level of evidence available in the literature is not high, it is advisable to continue ERT during pregnancy as there are several reports on safe continuation and delivery of healthy offspring of women on ERT during pregnancy.

One other concern for the use of ERT during pregnancy could be the potential drug related immune hypersensitivity reactions [90].

In addition to routine obstetric care, women with Pompe disease should be seen at least once every trimester by a specialist team. Anesthetic input should be discussed and arranged early, with local or regional anesthesia being the techniques of choice, while bearing in mind that muscular skeletal abnormalities may make this difficult. Discussions about mode of delivery and available options should be highlighted to the women, with patient involvement in the development of birth plans.

All investigations (baseline and subsequent) should be made available to obstetrics, neurology and anesthetic colleagues prior to assessment and birth-planning consultations. Input by obstetric, neurology, respiratory, anesthetic and dietician specialists (in addition to metabolic consultant specialist input) should be determined on an individual case basis depending on baseline and progressive symptoms, severity of disease and previous obstetric history.

The use of the home ventilator and the in-exsufflator in the perioperative period should be considered to avoid intubation.

It has been shown that ERT is secreted in low amounts in breast milk after an ERT infusion. Therefore, it is advised not to breastfeed children within the first 24 h after ERT infusion. If preferred, the mother may use previously expressed milk during the 24 h after the last infusion and discard expressed milk during this time [98].

## Supplementary Information

Additional file 1. Quality Assessment of references: References list evaluated based on the AGREE II criteria and Grading of Recommendations, Assessment, Development and Evaluation (GRADE) methodology.

## Data Availability

Data sharing not applicable to this article as no datasets were generated or analyzed during the current study.

## Abbreviations

4MUG: 4-Methylumbelliferyl-α-d-glucopyranoside
6-MWT: 6-Minute walking test
ABR/BAEP: Auditory evoked potentials
ACMG-AMP: American College of Medical Genetics and genomics and Association for Molecular Pathology
AIMS: Alberta Infant Motor Scale
ALT: Alanine aminotransferase
ARF: Acute respiratory failure
AST: Aspartate aminotransferase
BNP: Brain natriuretic peptide
CDG: Congenital defects of glycosylation
CK: Creatine kinase
CPR: Clinical pathway recommendations
CRIM: Cross reactive immune material
CT: Computed tomography
DEXA: Dual-energy X-ray absorptiometry
EMA: European Medicines Agency
EMG: Electromyography
EPoC: European Pompe disease Consortium
ERT: Enzyme replacement therapy
EU: European Union
FDA: Food and Drug Administration
FSS: Fatigue by the fatigue intensity scale
FVC: Forced vital capacity
GAA: Acid alpha-glucosidase
GL: Guidelines
Glc4: Glucose tetrasaccharide
GRADE: Grading of Recommendations, Assessment, Development and Evaluation
GYS1: Glycogen synthase
HCP: Healthcare providers
IOPD: Infantile-onset Pompe disease
LDH: Lactate dehydrogenase
LOPD: Late-onset Pompe disease
LSD-SNW: Lysosomal storage disease subnetwork
MIP/MEP: Maximun inspiratory pressure and maximum expiratory pressure
MMT-MRC: Muscular force by Medical Research Council
MRI: Magnetic resonance imaging
NBS: Newborn screening
NGS: Next generation sequencing
NPPV: Non-invasive positive pressure ventilation
PD: Pompe disease
PEDI: Pediatric evaluation of disability inventory
QoL: Quality of life
rhGAA: Recombinant human GAA
RHS: Rotterdam Handicap Scale
RICU: Respiratory intensive care unit
RSV: Respiratory syncytial virus
RUSP: Recommended universal screening panel
SF-36: Short form 36
VLCAD: Very long-chain acyl-CoA dehydrogenase
VUS: Variant of unknown significance

## References

[1] Shea L, Raben N. Autophagy in skeletal muscle: implications for Pompe disease. Int J Clin Pharmacol Ther. 2009;47(Suppl 1):42–7. 10.5414/cpp47042 PMC2948975 20040311

[2] Myerowitz R, Puertollano R, Raben N. Impaired autophagy: the collateral damage of lysosomal storage disorders. EBioMedicine. 2021;63:103166. 33341443 10.1016/j.ebiom.2020.103166 PMC7753127

[3] Lim JA, Li L, Kakhlon O, Myerowitz R, Raben N. Defects in calcium homeostasis and mitochondria can be reversed in Pompe disease. Autophagy. 2015;11(2):385–402. 25758767 10.1080/15548627.2015.1009779 PMC4502791

[4] Tarallo A, Damiano C, Strollo S, Minopoli N, Indrieri A, Polishchuk E, et al. Correction of oxidative stress enhances enzyme replacement therapy in Pompe disease. EMBO Mol Med. 2021;13(11):e14434. 10.15252/emmm.202114434. 34606154 10.15252/emmm.202114434 PMC8573602

[5] van der Ploeg AT, Laforet P. Pompe disease. In: Hollak C, Lachmann R, editors. Inherited metabolic disease in adults: a clinical guide. Oxford University Press; 2016. p. 353–8.

[6] Martiniuk F, Bodkin M, Tzall S, Hirschhorn R. Isolation and partial characterization of the structural gene for human acid alpha glucosidase. DNA Cell Biol. 1991;10(4):283–92. 1674202 10.1089/dna.1991.10.283

[7] van der Ploeg AT, Reuser AJ. Pompe’s disease. Lancet. 2008;372(9646):1342–53. 18929906 10.1016/S0140-6736(08)61555-X

[8] de Faria DOS, In’t Groen SLM, Hoogeveen-Westerveld M, Nino MY, van der Ploeg AT, Bergsma AJ, et al. Update of the Pompe variant database for the prediction of clinical phenotypes: novel disease-associated variants, common sequence variants, and results from newborn screening. Hum Mutat. 2021;42(2):119–34. 33560568 10.1002/humu.24148 PMC7898817

[9] Bergsma AJ, van den Dorpel JJ, van den Hout HJ, van der Beek NA, Schoser B, et al. A genetic modifier of symptom onset in Pompe disease. EBioMedicine. 2019;43:553–61. 30922962 10.1016/j.ebiom.2019.03.048 PMC6562017

[10] Musumeci O, Thieme A, Claeys KG, Wenninger S, Kley RA, Kuhn M, et al. Homozygosity for the common GAA gene splice site mutation c.-32–13T>G in Pompe disease is associated with the classical adult phenotypical spectrum. Neuromuscul Disord. 2015;25(9):719–24. 26231297 10.1016/j.nmd.2015.07.002

[11] Hermans MM, van Leenen D, Kroos MA, Beesley CE, Van Der Ploeg AT, Sakuraba H, et al. Twenty-two novel mutations in the lysosomal alpha-glucosidase gene (GAA) underscore the genotype-phenotype correlation in glycogen storage disease type II. Hum Mutat. 2004;23(1):47–56. 14695532 10.1002/humu.10286

[12] Reuser AJJ, van der Ploeg AT, Chien YH, Llerena J Jr, Abbott MA, Clemens PR, et al. On behalf of the Pompe registry sites. GAA variants and phenotypes among 1079 patients with Pompe disease: data from the Pompe registry. Hum Mutat. 2019;40(11):2146–64. 31342611 10.1002/humu.23878 PMC6852536

[13] Niño MY, Wijgerde M, de Faria DOS, Hoogeveen-Westerveld M, Bergsma AJ, Broeders M, et al. Enzymatic diagnosis of Pompe disease: lessons from 28 years of experience. Eur J Hum Genet. 2021;29(3):434–46. 33162552 10.1038/s41431-020-00752-2 PMC7940434

[14] van Gelder CM, Hoogeveen-Westerveld M, Kroos MA, Plug I, van der Ploeg AT, Reuser AJ. Enzyme therapy and immune response in relation to CRIM status: the Dutch experience in classic infantile Pompe disease. J Inherit Metab Dis. 2015;38(2):305–14. 24715333 10.1007/s10545-014-9707-6 PMC4341007

[15] Banugaria SG, Prater SN, Patel TT, Dearmey SM, Milleson C, Sheets KB, et al. Algorithm for the early diagnosis and treatment of patients with cross reactive immunologic material-negative classic infantile Pompe disease: a step towards improving the efficacy of ERT. PLoS ONE. 2013;8(6):e67052. 10.1371/journal.pone.0067052. 23825616 10.1371/journal.pone.0067052 PMC3692419

[16] Semplicini C, Letard P, De Antonio M, Taouagh N, Perniconi B, Bouhour F, French Pompe Study Group, et al. Late-onset Pompe disease in France: molecular features and epidemiology from a nationwide study. J Inherit Metab Dis. 2018;41(6):937–46. 30155607 10.1007/s10545-018-0243-7

[17] Scott CR, Elliott S, Buroker N, Thomas LI, Keutzer J, Glass M, et al. Identification of infants at risk for developing Fabry, Pompe, or mucopolysaccharidosis-I from newborn blood spots by tandem mass spectrometry. J Pediatr. 2013;163(2):498–503. 23465405 10.1016/j.jpeds.2013.01.031 PMC3725184

[18] Bodamer OA, Scott CR, Giugliani R. Pompe Disease Newborn Screening Working Group. Newborn screening for Pompe disease. Pediatrics. 2017;140(Suppl 1):4–13. 10.1542/peds.2016-0280C 29162673

[19] Chien YH, Hwu WL, Lee NC. Newborn screening: Taiwanese experience. Ann Transl Med. 2019;7(13):281. 31392193 10.21037/atm.2019.05.47 PMC6642927

[20] Gragnaniello V, Pijnappel PWWM, Burlina AP, In’t-Groen SLM, Gueraldi D, Cazzorla C, et al. Newborn screening for Pompe disease in Italy: long-term results and future challenges. Mol Genet Metab Rep. 2022;33:100929. 36310651 10.1016/j.ymgmr.2022.100929 PMC9597184

[21] Mechtler TP, Stary S, Metz TF, De Jesús VR, Greber-Platzer S, Pollak A, et al. Neonatal screening for lysosomal storage disorders: feasibility and incidence from a nationwide study in Austria. Lancet. 2012;379(9813):335–41. 22133539 10.1016/S0140-6736(11)61266-X

[22] Elenga N, Verloes A, Mrsic Y, Basurko C, Schaub R, Cuadro-Alvarez E, et al. Incidence of infantile Pompe disease in the Maroon population of French Guiana. BMJ Paediatr Open. 2018;2(1):e000182. 10.1136/bmjpo-2017-000182. 29637184 10.1136/bmjpo-2017-000182 PMC5842995

[23] Hagemans ML, Winkel LP, Hop WC, Reuser AJ, Van Doorn PA, Van der Ploeg AT. Disease severity in children and adults with Pompe disease related to age and disease duration. Neurology. 2005;64(12):2139–41. 15985590 10.1212/01.WNL.0000165979.46537.56

[24] Kroos MA, Pomponio RJ, Hagemans ML, Keulemans JL, Phipps M, DeRiso M, et al. Broad spectrum of Pompe disease in patients with the same c.-32–13T->G haplotype. Neurology. 2007;68(2):110–5. 17210890 10.1212/01.wnl.0000252798.25690.76

[25] Preisler N, Lukacs Z, Vinge L, Madsen KL, Husu E, Hansen RS, et al. Late-onset Pompe disease is prevalent in unclassified limb-girdle muscular dystrophies. Mol Genet Metab. 2013;110(3):287–9. 24011652 10.1016/j.ymgme.2013.08.005

[26] Sawada T, Kido J, Nakamura K. Newborn screening for Pompe disease. Int J Neonatal Screen. 2020;6(2):31. 33073027 10.3390/ijns6020031 PMC7423004

[27] Smith LD, Bainbridge MN, Parad RB, Bhattacharjee A. Second tier molecular genetic testing in newborn screening for pompe disease: landscape and challenges. Int J Neonatal Screen. 2020;6(2):32. 32352041 10.3390/ijns6020032 PMC7189780

[28] van El CG, Rigter T, Reuser AJ, van der Ploeg AT, Weinreich SS, Cornel MC. Newborn screening for pompe disease? A qualitative study exploring professional views. BMC Pediatr. 2014;14:203. 25124044 10.1186/1471-2431-14-203 PMC4139142

[29] Weinreich SS, Rigter T, van El CG, Dondorp WJ, Kostense PJ, van der Ploeg AT, et al. Public support for neonatal screening for Pompe disease, a broad-phenotype condition. Orphanet J Rare Dis. 2012;7:15. 22413814 10.1186/1750-1172-7-15 PMC3351372

[30] Singh S, Ojodu J, Kemper AR, Lam WKK, Grosse SD. Implementation of newborn screening for conditions in the United States first recommended during 2010–2018. Int J Neonatal Screen. 2023;9(2):20. 37092514 10.3390/ijns9020020 PMC10123615

[31] Gelb MH, Turecek F, Scott CR, Chamoles NA. Direct multiplex assay of enzymes in dried blood spots by tandem mass spectrometry for the newborn screening of lysosomal storage disorders. J Inherit Metab Dis. 2006;29(2–3):397–404. 16763908 10.1007/s10545-006-0265-4 PMC2488386

[32] Labrousse P, Chien YH, Pomponio RJ, Keutzer J, Lee NC, Akmaev VR, et al. Genetic heterozygosity and pseudodeficiency in the Pompe disease newborn screening pilot program. Mol Genet Metab. 2010;99(4):379–83. 20080426 10.1016/j.ymgme.2009.12.014

[33] Kroos M, Hoogeveen-Westerveld M, van der Ploeg A, Reuser AJ. The genotype-phenotype correlation in Pompe disease. Am J Med Genet C Semin Med Genet. 2012;160C(1):59–68. 22253258 10.1002/ajmg.c.31318

[34] van der Ploeg AT, Kruijshaar ME, Toscano A, Laforêt P, Angelini C, Lachmann RH, European Pompe Consortium, et al. European consensus for starting and stopping enzyme replacement therapy in adult patients with Pompe disease: a 10-year experience. Eur J Neurol. 2017;24(6):768. 10.1111/ene.13285. 28477382 10.1111/ene.13285

[35] Pompe Disease Diagnostic Working Group, Winchester B, Bali D, Bodamer OA, Caillaud C, Christensen E, et al. Methods for a prompt and reliable laboratory diagnosis of Pompe disease: report from an international consensus meeting. Mol Genet Metab. 2008;93(3):275–81. 18078773 10.1016/j.ymgme.2007.09.006

[36] Savarese M, Torella A, Musumeci O, Angelini C, Astrea G, Bello L, et al. Targeted gene panel screening is an effective tool to identify undiagnosed late onset Pompe disease. Neuromuscul Disord. 2018;28(7):586–91. 29880332 10.1016/j.nmd.2018.03.011

[37] In’t Groen SLM, de Faria DOS, Iuliano A, van den Hout JMP, Douben H, Dijkhuizen T, et al. Novel GAA variants and mosaicism in Pompe disease identified by extended analyses of patients with an incomplete DNA diagnosis. Mol Ther Methods Clin Dev. 2020;17:337–48. 32071926 10.1016/j.omtm.2019.12.016 PMC7013133

[38] Pascarella A, Terracciano C, Farina O, Lombardi L, Esposito T, Napolitano F, et al. Vacuolated PAS-positive lymphocytes as an hallmark of Pompe disease and other myopathies related to impaired autophagy. J Cell Physiol. 2018;233(8):5829–37. 29215735 10.1002/jcp.26365

[39] Hahn A, Hennermann JB, Huemer M, Kampmann C, Marquardt T, Mengel E, et al. Diagnosis and care of infants and children with Pompe disease. Klin Padiatr. 2020. 10.1055/a-1110-7335. 32069498 10.1055/a-1110-7335

[40] Saville JT, Fuller M. Experience with the urinary tetrasaccharide metabolite for Pompe disease in the diagnostic laboratory. Metabolites. 2021;11(7):446. 34357340 10.3390/metabo11070446 PMC8305466

[41] Tarallo A, Carissimo A, Gatto F, Nusco E, Toscano A, Musumeci O, et al. microRNAs as biomarkers in Pompe disease. Genet Med. 2019;21(3):591–600. 29997386 10.1038/s41436-018-0103-8

[42] Carrasco-Rozas A, Fernández-Simón E, Lleixà MC, Belmonte I, Pedrosa-Hernandez I, Montiel-Morillo E, et al. Identification of serum microRNAs as potential biomarkers in Pompe disease. Ann Clin Transl Neurol. 2019;6(7):1214–24. 31353854 10.1002/acn3.50800 PMC6649638

[43] Mackenbach MJ, Willemse EAJ, van den Dorpel JJA, van der Beek NAME, Díaz-Manera J, Rizopoulos D, et al. Neurofilament light and its association with CNS involvement in patients with classic infantile Pompe disease. Neurology. 2023;101(6):e594–601. 10.1212/WNL.0000000000207482. 37336766 10.1212/WNL.0000000000207482 PMC10424841

[44] Hsu YK, Chien YH, Shinn-Forng Peng S, Hwu WL, Lee WT, Lee NC, et al. Evaluating brain white matter hyperintensity, IQ scores, and plasma neurofilament light chain concentration in early-treated patients with infantile-onset Pompe disease. Genet Med. 2023;25(1):27–36. 36399131 10.1016/j.gim.2022.10.005

[45] Harlaar L, Ciet P, van Tulder G, Brusse E, Timmermans RGM, Janssen WGM, et al. Diaphragmatic dysfunction in neuromuscular disease, an MRI study. Neuromuscul Disord. 2022;32(1):15–24. 34973872 10.1016/j.nmd.2021.11.001

[46] Harlaar L, Ciet P, van der Ploeg AT, Brusse E, van der Beek NAME, Wielopolski PA, et al. Imaging of respiratory muscles in neuromuscular disease: a review. Neuromuscul Disord. 2018;28(3):246–56. 29398294 10.1016/j.nmd.2017.11.010

[47] Ebbink BJ, Poelman E, Aarsen FK, Plug I, Régal L, Muentjes C, et al. Classic infantile Pompe patients approaching adulthood: a cohort study on consequences for the brain. Dev Med Child Neurol. 2018;60(6):579–86. 29573408 10.1111/dmcn.13740

[48] Labella B, Cotti Piccinelli S, Risi B, Caria F, Damioli S, Bertella E, et al. A comprehensive update on late-onset Pompe disease. Biomolecules. 2023;13(9):1279. 37759679 10.3390/biom13091279 PMC10526932

[49] Van den Hout JM, Kamphoven JH, Winkel LP, Arts WF, De Klerk JB, Loonen MC, et al. Long-term intravenous treatment of Pompe disease with recombinant human alpha-glucosidase from milk. Pediatrics. 2004;113(5):e448–57. 10.1542/peds.113.5.e448. 15121988 10.1542/peds.113.5.e448

[50] van der Ploeg AT, Clemens PR, Corzo D, Escolar DM, Florence J, Groeneveld GJ, et al. A randomized study of alglucosidase alfa in late-onset Pompe’s disease. N Engl J Med. 2010;362(15):1396–406. 20393176 10.1056/NEJMoa0909859

[51] Kuperus E, Kruijshaar ME, Wens SCA, de Vries JM, Favejee MM, van der Meijden JC, et al. Long-term benefit of enzyme replacement therapy in Pompe disease: a 5-year prospective study. Neurology. 2017;89(23):2365–73. 29117951 10.1212/WNL.0000000000004711

[52] Gutschmidt K, Musumeci O, Díaz-Manera J, Chien YH, Knop KC, Wenninger S, et al. STIG study: real-world data of long-term outcomes of adults with Pompe disease under enzyme replacement therapy with alglucosidase alfa. J Neurol. 2021;268(7):2482–92. 33543425 10.1007/s00415-021-10409-9 PMC7862044

[53] Ditters IAM, Huidekoper HH, Kruijshaar ME, Rizopoulos D, Hahn A, Mongini TE, European Pompe Consortium Project Group on Classic Infantile Pompe Disease, et al. European Pompe Consortium project group on classic infantile Pompe disease. Effect of alglucosidase alfa dosage on survival and walking ability in patients with classic infantile Pompe disease: a multicentre observational cohort study from the European Pompe Consortium. Lancet Child Adolesc Health. 2022;6(1):28–37. 34822769 10.1016/S2352-4642(21)00308-4

[54] Dalmia S, Sharma R, Ramaswami U, Hughes D, Jahnke N, Cole D, et al. Enzyme replacement therapy for late-onset Pompe disease. Cochrane Database Syst Rev. 2023;12(12):CD012993. 10.1002/14651858.CD012993.pub2. 38084761 10.1002/14651858.CD012993.pub2 PMC10714667

[55] Diaz-Manera J, Kishnani PS, Kushlaf H, Ladha S, Mozaffar T, Straub V, COMET Investigator Group, et al. Safety and efficacy of avalglucosidase alfa versus alglucosidase alfa in patients with late-onset Pompe disease (COMET): a phase 3, randomised, multicentre trial. Lancet Neurol. 2021;20(12):1012–26. 34800399 10.1016/S1474-4422(21)00241-6

[56] Schoser B, Roberts M, Byrne BJ, Sitaraman S, Jiang H, Laforêt P, PROPEL Study Group, et al. Safety and efficacy of cipaglucosidase alfa plus miglustat versus alglucosidase alfa plus placebo in late-onset Pompe disease (PROPEL): an international, randomised, double-blind, parallel-group, phase 3 trial. Lancet Neurol. 2021;20(12):1027–37. 34800400 10.1016/S1474-4422(21)00331-8

[57] Blair HA. Cipaglucosidase Alfa: first approval. Drugs. 2023;83(8):739–45. 37184753 10.1007/s40265-023-01886-5 PMC10184071

[58] Poelman E, van den Dorpel JJA, Hoogeveen-Westerveld M, van den Hout JMP, van der Giessen LJ, van der Beek NAME, et al. Effects of higher and more frequent dosing of alglucosidase alfa and immunomodulation on long-term clinical outcome of classic infantile Pompe patients. J Inherit Metab Dis. 2020;43(6):1243–53. 32506446 10.1002/jimd.12268 PMC7689828

[59] Khan AA, Case LE, Herbert M, DeArmey S, Jones H, Crisp K, et al. Higher dosing of alglucosidase alfa improves outcomes in children with Pompe disease: a clinical study and review of the literature. Genet Med. 2020;22(5):898–907. 31904026 10.1038/s41436-019-0738-0 PMC7469631

[60] Chien YH, Tsai WH, Chang CL, Chiu PC, Chou YY, Tsai FJ, et al. Earlier and higher dosing of alglucosidase alfa improve outcomes in patients with infantile-onset Pompe disease: evidence from real-world experiences. Mol Genet Metab Rep. 2020;23:100591. 32373469 10.1016/j.ymgmr.2020.100591 PMC7193123

[61] Dhillon S. Avalglucosidase alfa: first approval. Drugs. 2021;81(15):1803–9. 34591286 10.1007/s40265-021-01600-3

[62] Kishnani PS, Diaz-Manera J, Toscano A, Clemens PR, Ladha S, Berger KI, COMET Investigator Group, et al. Efficacy and safety of avalglucosidase alfa in patients with late-onset pompe disease after 97 weeks: a phase 3 randomized clinical trial. JAMA Neurol. 2023;80(6):558–67. 37036722 10.1001/jamaneurol.2023.0552 PMC10087094

[63] Kishnani PS, Kronn D, Brassier A, Broomfield A, Davison J, Hahn SH, Mini-COMET Investigators, et al. Safety and efficacy of avalglucosidase alfa in individuals with infantile-onset Pompe disease enrolled in the phase 2, open-label Mini-COMET study: the 6-month primary analysis report. Genet Med. 2023;25(2):100328. 36542086 10.1016/j.gim.2022.10.010

[64] Schoser B, Stewart A, Kanters S, Hamed A, Jansen J, Chan K, et al. Survival and long-term outcomes in late-onset Pompe disease following alglucosidase alfa treatment: a systematic review and meta-analysis. J Neurol. 2017;264(4):621–30. 27372449 10.1007/s00415-016-8219-8

[65] Yang CF, Yang CC, Liao HC, Huang LY, Chiang CC, Ho HC, et al. Very early treatment for infantile-onset pompe disease contributes to better outcomes. J Pediatr. 2016;169:174-80.e1. 10.1016/j.jpeds.2015.10.078. 26685070 10.1016/j.jpeds.2015.10.078

[66] Schoser B, van der Beek NAME, Broomfield A, Brusse E, Diaz-Manera J, Hahn A, et al. Start, switch and stop (triple-S) criteria for enzyme replacement therapy of late-onset Pompe disease: European Pompe Consortium recommendation update 2024. Eur J Neurol. 2024:e16383. 10.1111/ene.16383 PMC11295151 38873957

[67] Ditters IAM, van der Beek NAME, Brusse E, van der Ploeg AT, van den Hout JMP, Huidekoper HH. Home-based enzyme replacement therapy in children and adults with Pompe disease; a prospective study. Orphanet J Rare Dis. 2023;18(1):108. 37158969 10.1186/s13023-023-02715-4 PMC10169363

[68] Ditters IAM, van Kooten HA, van der Beek NAME, Hardon JF, Ismailova G, Brusse E, et al. Home-based infusion of alglucosidase alfa can safely be implemented in adults with late-onset Pompe disease: lessons learned from 18,380 infusions. BioDrugs. 2023;37(5):685–98. 37326923 10.1007/s40259-023-00609-2 PMC10432339

[69] Kishnani PS, Dickson PI, Muldowney L, Lee JJ, Rosenberg A, Abichandani R, et al. Immune response to enzyme replacement therapies in lysosomal storage diseases and the role of immune tolerance induction. Mol Genet Metab. 2016;117(2):66–83. 26597321 10.1016/j.ymgme.2015.11.001

[70] Poelman E, Hoogeveen-Westerveld M, van den Hout JMP, van den Hout JMP, Bredius RGM, Lankester AC, et al. Effects of immunomodulation in classic infantile Pompe patients with high antibody titers. Orphanet J Rare Dis. 2019;14(1):71. 30902109 10.1186/s13023-019-1039-z PMC6431009

[71] Li C, Desai AK, Gupta P, Dempsey K, Bhambhani V, Hopkin RJ, et al. Transforming the clinical outcome in CRIM-negative infantile Pompe disease identified via newborn screening: the benefits of early treatment with enzyme replacement therapy and immune tolerance induction. Genet Med. 2021;23(5):845–55. 33495531 10.1038/s41436-020-01080-y PMC8107133

[72] Deodato F, Ginocchio VM, Onofri A, Grutter G, Germani A, Dionisi-Vici C. Immune tolerance induced using plasma exchange and rituximab in an infantile Pompe disease patient. J Child Neurol. 2014;29(6):850–4. 23620524 10.1177/0883073813485819

[73] Desai AK, Rosenberg AS, Kishnani PS. The potential impact of timing of IVIG administration on the efficacy of rituximab for immune tolerance induction for patients with Pompe disease. Clin Immunol. 2020;219:108541. 32681978 10.1016/j.clim.2020.108541

[74] Desai AK, Shrivastava G, Grant CL, Wang RY, Burt TD, Kishnani PS. An updated management approach of Pompe disease patients with high-sustained anti-rhGAA IgG antibody titers: experience with bortezomib-based immunomodulation. Front Immunol. 2024;15:1360369. 38524130 10.3389/fimmu.2024.1360369 PMC10959098

[75] El-Gharbawy AH, Mackey J, DeArmey S, Westby G, Grinnell SG, Malovrh P, et al. An individually, modified approach to desensitize infants and young children with Pompe disease, and significant reactions to alglucosidase alfa infusions. Mol Genet Metab. 2011;104(1–2):118–22. 21802969 10.1016/j.ymgme.2011.07.004 PMC3711228

[76] Gragnaniello V, Fecarotta S, Pecoraro A, Tarallo A, Catzola A, Spadaro G, et al. Desensitization of two young patients with infantile-onset Pompe disease and severe reactions to alglucosidase alfa. Neurol Sci. 2019;40(7):1453–5. 30778879 10.1007/s10072-019-03744-3

[77] Koeberl DD, Case LE, Desai A, Smith EC, Walters C, Han SO, et al. Improved muscle function in a phase I/II clinical trial of albuterol in Pompe disease. Mol Genet Metab. 2020;129(2):67–72. 31839530 10.1016/j.ymgme.2019.12.008

[78] ClinicalTrial.gov. Clinical Trial Identifier NCT01942590

[79] ClinicalTrial.gov. Clinical Trial Identifier NCT01885936

[80] Byrne BJ, Falk DJ, Pacak CA, Nayak S, Herzog RW, Elder ME, et al. Pompe disease gene therapy. Hum Mol Genet. 2011;20(R1):R61–8. 10.1093/hmg/ddr174. 21518733 10.1093/hmg/ddr174 PMC3095055

[81] Colella P, Mingozzi F. Gene therapy for pompe disease: the time is now. Hum Gene Ther. 2019;30(10):1245–62. 31298581 10.1089/hum.2019.109

[82] Ullman JC, Mellem KT, Xi Y, Ramanan V, Merritt H, Choy R, et al. Small-molecule inhibition of glycogen synthase 1 for the treatment of Pompe disease and other glycogen storage disorders. Sci Transl Med. 2024;16(730):eadf1691. 10.1126/scitranslmed.adf1691. 38232139 10.1126/scitranslmed.adf1691 PMC10962247

[83] Carubbi F, Barbato A, Burlina AB, Francini F, Mignani R, Pegoraro E, Italian Society of Human Nutrition Working Group on Nutrition in Lysosomal Storage Diseases, et al. Nutrition in adult patients with selected lysosomal storage diseases. Nutr Metab Cardiovasc Dis. 2021;31(3):733–44. 33589321 10.1016/j.numecd.2020.11.028

[84] Morales JA, Anilkumar AC. Glycogen Storage Disease Type II. In: StatPearls [Internet]. Treasure Island (FL): StatPearls Publishing; 2023. https://www.statpearls.com/point-of-care/22333. Accessed 16 Jul 2024 29262159

[85] Favejee MM, Huisstede BM, Bussmann JB, Kruijshaar ME, van der Ploeg AT. Physiotherapy management in late-onset Pompe disease: clinical practice in 88 patients. Mol Genet Metab. 2012;107(1–2):111–5. 22901700 10.1016/j.ymgme.2012.07.014

[86] Favejee MM, van den Berg LE, Kruijshaar ME, Wens SC, Praet SF, Pim Pijnappel WW, et al. Exercise training in adults with Pompe disease: the effects on pain, fatigue, and functioning. Arch Phys Med Rehabil. 2015;96(5):817–22. 25499687 10.1016/j.apmr.2014.11.020

[87] Mellies U, Stehling F, Dohna-Schwake C, Ragette R, Teschler H, Voit T. Respiratory failure in Pompe disease: treatment with noninvasive ventilation. Neurology. 2005;64(8):1465–7. 15851748 10.1212/01.WNL.0000158682.85052.C0

[88] Boentert M, Prigent H, Várdi K, Jones HN, Mellies U, Simonds AK, et al. Practical recommendations for diagnosis and management of respiratory muscle weakness in late-onset Pompe disease. Int J Mol Sci. 2016;17(10):1735. 27763517 10.3390/ijms17101735 PMC5085764

[89] Bembi B, Cerini E, Danesino C, Donati MA, Gasperini S, Morandi L, et al. Management and treatment of glycogenosis type II. Neurology. 2008;71(23_Suppl 2):12–36. 10.1212/WNL.0b013e31818da93f 19047571

[90] Kishnani PS, Steiner RD, Bali D, Berger K, Byrne BJ, Case LE, et al. Pompe disease diagnosis and management guideline. Genet Med. 2006;8(5):267–88 (**Erratum in: Genet Med. 2006;8(6):382. ACMG Work Group on Management of Pompe Disease**). 16702877 10.1097/01.gim.0000218152.87434.f3 PMC3110959

[91] Leslie N, Bailey L. Pompe Disease. In: Adam MP, Feldman J, Mirzaa GM, Pagon RA, Wallace SE, Bean LJH, Gripp KW, Amemiya A, editors. GeneReviews^®^ [Internet]. Seattle (WA): University of Washington, Seattle; 1993–2024. 2007 Aug 31 [updated 2023 Nov 2]. https://www.ncbi.nlm.nih.gov/books/NBK1261/20301438

[92] PGüngör D, Kruijshaar ME, Plug I, Rizopoulos D, Kanters TA, Wens SC, et al. Quality of life and participation in daily life of adults with Pompe disease receiving enzyme replacement therapy: 10 years of international follow-up. J Inherit Metab Dis. 2016;39(2):253–60. 26531313 10.1007/s10545-015-9889-6 PMC4754323

[93] Korlimarla A, Spiridigliozzi GA, Stefanescu M, Austin SL, Kishnani PS. Behavioral, social and school functioning in children with Pompe disease. Mol Genet Metab Rep. 2020;25:100635. 32793419 10.1016/j.ymgmr.2020.100635 PMC7414001

[94] Angelini C, Siciliano G. Neuromuscular diseases and Covid-19: advices from scientific societies and early observations in Italy. Eur J Transl Myol. 2020;30(2):9032. 32782765 10.4081/ejtm.2019.9032 PMC7385692

[95] Plöckinger U, Tiling N, Bosanska L, Temmesfeld-Wollbrueck B, Irlbacher K, Mezger V, et al. Multiple, successful pregnancies in Pompe disease. JIMD Rep. 2016;28:111–8. 26572913 10.1007/8904_2015_518 PMC5059215

[96] Rohman PJ, Scott E, Richfield L, Ramaswami U, Hughes DA. Pregnancy and associated events in women receiving enzyme replacement therapy for late-onset glycogen storage disease type II (Pompe disease). J Obstet Gynaecol Res. 2016;42(10):1263–71. 27384519 10.1111/jog.13055

[97] Goker-Alpan O, Kasturi VG, Sohi MK, Limgala RP, Austin SL, Jennelle T, et al. Pregnancy outcomes in late onset pompe disease. Life. 2020;10(9):194. 32932790 10.3390/life10090194 PMC7556025

[98] de Vries JM, Brugma JD, Ozkan L, Steegers EA, Reuser AJ, van Doorn PA, et al. First experience with enzyme replacement therapy during pregnancy and lactation in Pompe disease. Mol Genet Metab. 2011;104(4):552–5. 21967859 10.1016/j.ymgme.2011.09.012

